# Analysis of Food Habits during Pandemic in a Polish Population-Based Sample of Primary School Adolescents: Diet and Activity of Youth during COVID-19 (DAY-19) Study

**DOI:** 10.3390/nu13113711

**Published:** 2021-10-22

**Authors:** Aleksandra Kołota, Dominika Głąbska

**Affiliations:** Department of Dietetics, Institute of Human Nutrition Sciences, Warsaw University of Life Sciences (SGGW-WULS), 159c Nowoursynowska Street, 02-776 Warsaw, Poland; dominika_glabska@sggw.edu.pl

**Keywords:** adolescents, body mass, obesity, diet, physical activity, food habits, Adolescents’ Food Habits Checklist (AFHC), COVID-19 pandemic, DAY-19 study

## Abstract

The improper dietary behaviors of children and adolescents during the COVID-19 pandemic, which are associated with lockdowns and reduced physical activity, are a complex problem, potentially resulting in increased risk of diet-related diseases, including overweight and obesity and their consequences. The aim of the study was to assess the food habits during the COVID-19 pandemic and to define their association with physical activity and body mass changes in a Polish population of primary school adolescents within the Diet and Activity of Youth During COVID-19 (DAY-19) Study. The DAY-19 Study was conducted in June 2020 in a national cohort of 1334 primary school students aged 10–16 years, recruited based on a stratified random sampling of schools (sampling counties from voivodeships and schools from counties). The Adolescent Food Habits Checklist (AFHC) was used to assess food habits, associated with food purchase, preparation, and consumption, which in the studied group were analyzed separately for the period before (retrospective data) and during the COVID-19 pandemic (prospective data). The recognizable physical activity changes and recognizable body mass changes were also assessed (retrospective data) and respondents were classified as those declaring that their physical activity and body mass decreased, remained stable, or increased during the COVID-19 pandemic. It was observed that during the COVID-19 pandemic the majority of food habits changed in a statistically significant way (*p* < 0.05). Within food purchase habits, the number of respondents who declared choosing a low-fat lunch away from home decreased, often buying pastries or cakes decreased, and buying a low-fat crisps brand increased (*p* < 0.05). Within food preparation habits, the number of respondents who declared trying to keep overall fat intake down increased, trying to keep overall sugar intake down increased, eating at least one serving of vegetables or salad with evening meal increased, and usually including some chocolate and/or biscuits in a packed lunch decreased (*p* < 0.05). Within food consumption habits, the number of respondents who declared making sure that they eat at least one serving of fruit a day increased, eating at least three servings of fruit most days increased, making sure that they eat at least one serving of vegetables or salad a day increased, trying to ensure that they eat plenty of fruit and vegetables increased, often choosing a fruit when they have a snack between meals increased, eating at least three servings of fruit most days increased, and generally trying to have a healthy diet increased (*p* < 0.05). It was concluded that in the period of the COVID-19 pandemic and resultant remote education, adolescents in Poland presented different food habits than before, while the majority of changes were positive. The positive food purchase, preparation, and consumption habits were observed mainly in sub-groups of adolescents declaring decreased body mass or increased physical activity during the COVID-19 pandemic. It may be suggested that physical activity may support positive changes of dietary behaviors and while combined positive changes of diet and increased physical activity, they may effectively promote body mass reduction in adolescents.

## 1. Introduction

The puberty period is crucial for the development of organisms due to key changes of anatomy, physiology, neurology, and behavior [1]. In this period, food preferences and food habits are created and strengthened, while changing from those characteristics for childhood to those stable through adulthood [2]. As a result, influencing food habits in the period of adolescence is a unique possibility to reduce the risk of diet-related health problems in adulthood [3]. A diversified and balanced diet enables proper development and growth of an organism, increased physical and mental functioning, and maintaining a proper body mass [4,5].

Especially important are fruit and vegetables, being directly associated with decreased risk of non-communicable diseases, including obesity [6,7], but also cardiovascular diseases [8], diabetes [9], and cancer in adulthood [10,11]. Moreover, fruit and vegetables’ intake in adolescence influences mental health [12] and self-assessment of health, including mental health [13]. As a result, the World Health Organization (WHO) emphasizes that a lot of countries recommended for adolescents to consume at least 400 g of fruit and vegetables per day [3], but a number of adolescents do not meet this recommendation [14] and in some countries their intake is decreasing, as was stated for fruit consumption in Germany, Greenland, Greece, Poland, and Portugal, as well as for vegetable consumption in Germany, Lithuania, Latvia, Poland, and Russia [15]. The indicated improper dietary habits in adolescents are not only a predictor of improper dietary habits in adults [16], but also are associated with excessive body mass [17], while following dietary recommendations protects from this [18].

The current situation of the outbreak of Coronavirus Disease 2019 (COVID-19) pandemic, which was associated with lockdowns and social isolation in numerous studies, also caused changes of nutritional behaviors, including shopping behaviors, that were observed both for adults [19,20], children, and adolescents [21,22]. However, the results of the studies conducted so far are not congruent, as, depending on the studied population, the research conducted so far revealed either positive or negative changes of eating behaviors. A study conducted in Spain indicated negative changes, including a decrease of fruit and vegetables’ intake in children and adolescents [21]. A study conducted in Brazil indicated positive changes, including reduced intake of unhealthy processed food products by families during their isolation [23]. At the same time, another study conducted in several countries including Brazil (Italy, Spain, Chile, Colombia, and Brazil) indicated positive changes resulting from more time spent by families on home cooking, but for adolescents no positive changes were observed [24].

Simultaneously, the COVID-19 pandemic reduced for children and adolescents the possibilities of physical activity and team sports’ practicing, due to remote learning systems as well as closed playgrounds and courts, which caused, in a number of studies, a reduced physical activity level in this population group [25,26,27,28]. However, in a number of studies conducted in a population of adolescents, while sport activities were decreased, habitual physical activities and outdoor activities, such as gardening, housework, cycling, skiing, or walking, were increased [29,30]. Such a situation results from the shift of physical activity from team sports and indoor sport activities to individual sports and outdoor sport activities, being imposed by legal regulations associated with the COVID-19 pandemic [31], but such an approach sometimes requires the commitment of parents [32]. As reduced physical activity in children and adolescents is commonly associated with worse nutritional habits [33,34], it should be indicated as a complex problem, potentially resulting in increased risk of diet-related diseases [35], including overweight and obesity [36]. Taking this into account, the food habits of children and adolescents during the COVID-19 pandemic are becoming a serious public health issue to be addressed.

Among various tools used to assess food habits, one of the commonly used questionnaires is the Adolescent Food Habits Checklist (AFHC) by Johnson et al. [37], which is used not only for adolescents [38,39] but also for adults [40]. This questionnaire was already used to assess the changes of food habits during the COVID-19 pandemic in the population of older adolescents [41,42], and studies revealed that while some food habits were improved, the other ones were worsened, which enabled defining essential areas for public health education [41]. In populations of younger adolescents [37,38], or even children [39], this tool was also used in multiple studies, but it has not been used so far in this population during the COVID-19 pandemic to assess their food habits changes.

Taking into consideration the issues described above, the aim of this study was to assess food habits during the COVID-19 pandemic and to define their association with physical activity and body mass changes in a Polish population of primary school adolescents within the Diet and Activity of Youth During COVID-19 (DAY-19) Study.

## 2. Materials and Methods

### 2.1. Ethical Statement

The conducted study was approved by the Ethics Committee of the Institute of Human Nutrition Sciences of the Warsaw University of Life Sciences (no. 18/2020). Within the study, the diet and physical activity were assessed [43] and their associations with body mass changes were analyzed.

All the procedures were based on the recommendations of the Declaration of Helsinki. From the participants of the study and their parents/legal guardians, their informed consent for the study participation was obtained. 

### 2.2. The Population Studied within DAY-19 Study

The DAY-19 Study was conducted in a Polish national cohort of primary school students aged 10–16 years. The participants of the study were recruited in June 2020, based on a stratified random sampling of schools, which was conducted in two phases, including: (1) sampling of counties from voivodeships (basic administrative units of Poland) and (2) sampling of schools from counties, which was conducted while using the Polish register of primary schools. The procedure was in agreement with the general approach applied in Poland to obtain a representative sample of students from all regions of the country, chosen especially during the COVID-19 pandemic [44,45]. In the first phase, 10 counties were sampled from each voivodeship (10 counties × 16 voivodeships) and in the second phase, 10 primary schools were sampled from each county (10 schools × 160 counties). All the sampled schools were invited to participate in the study, while the principal of each of them received a written invitation, accompanied with detailed protocol and all the information. If principals expressed the will for the school to participate in the study, they informed students that they were invited to participate, while their participation was also voluntary. The students were included only if they, as well as their parents/legal guardians, provided informed consent to participate. If so, they were provided an electronic link to the dedicated questionnaire which did not allow the identification of the study participants and did not gather any personal or sensitive data, except for age and the attended primary school.

All the students were encouraged to participate in the study, but similarly as for schools, a low response rate was observed. The participation in the study was voluntary both for schools and within the schools for students. The final number of participating schools was 43, and they represented all the regions of Poland.

The inclusion criteria for the participants of the study were as follows:

− Being a student of the sampled school;
− Being 10–16 years old;
− Informed consent for the study participation provided from both students and their parents/legal guardians.

No exclusion criteria based on the health status of participants were formulated, which was associated with the fact that the aim of the study was to assess general food habits during the COVID-19 pandemic in a population of Polish adolescents, independently from the presence of diet-related diseases. 

After collecting the completed questionnaires, some of them were excluded from the analysis, based on the following criteria:

− Any missing data in the provided questionnaire;
− Any unreliable data in the provided questionnaire (as in the other research [46], the data was considered to be unreliable if the respondent provided uniform answers across all of the questions, which was associated with failing to differentiate between answer choices, as they provided identical responses to all questions).

**Figure 1:**
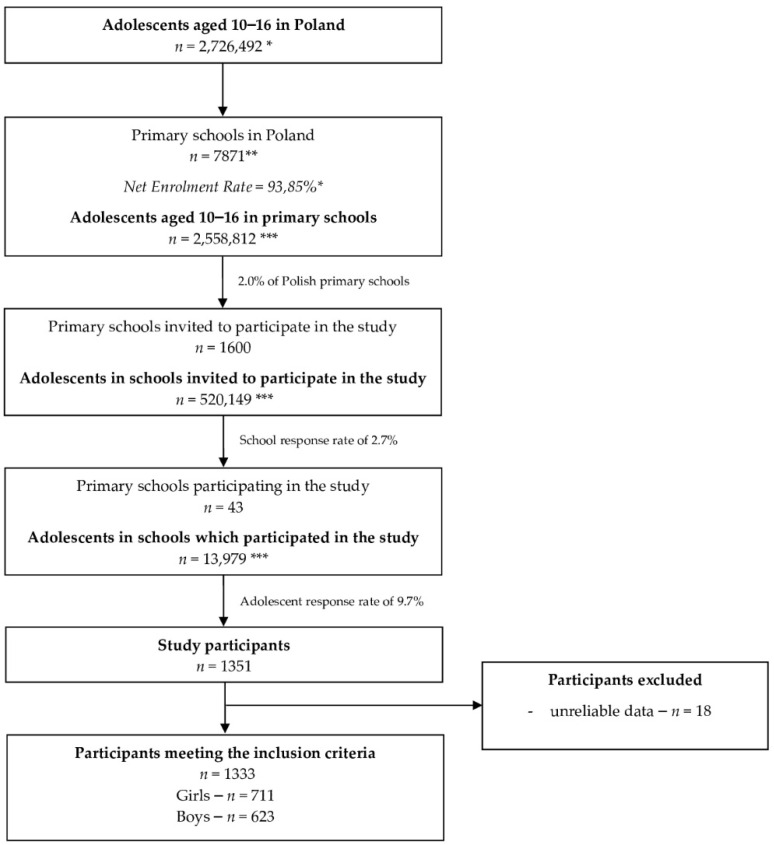
The recruitment procedure on flow chart for the adolescents from the Diet and Activity of Youth during COVID-19 (DAY-19) Study cohort (*n* = 1333); * [47,48] ** [49] *** calculated based on data of the Central Statistical Office.

The final sample of 1334 students participated in the study. The recruitment procedure is presented on flow chart (Figure 1).

### 2.3. Applied Questionnaire

The applied questionnaire included basic questions about recognizable body mass changes during the COVID-19 pandemic and recognizable physical activity changes during the COVID-19 pandemic, as well as included the AFHC by Johnson et al. [37], which is validated in a population of adolescents [37]. As AFHC is a questionnaire developed to be completed by adolescents, all the information was self-reported.

In the period when the study was conducted, based on the decision of the Polish Ministry of National Education, in Poland the system of remote learning was implemented [50], so in order to reflect properly changes of food habits during the COVID-19 pandemic, participants of the study were asked to describe separately their food habits before the period of remote learning (being the period before the COVID-19 pandemic) and current food habits (in the period of remote learning). Similarly, as in the previous Polish studies conducted in the population of adolescents [41], the question was formulated based on ensuring the period before implementing remote learning and the period of remote learning were easily recognizable for adolescents, as for adolescents the period of COVID-19 pandemic itself may be not so easy to define.

The AFHC [37] is based on 23 questions about various food habits, which are associated with food purchase, preparation, and consumption. The Polish version of AFHC was applied as it is commonly used [41,42], and was developed based on the forward and backward translation and transcultural adaptation [41]. 

The question about recognizable physical activity changes during the COVID-19 pandemic was based on the subjective assessment of participants. They were asked to declare if their physical activity decreased, remained stable, or increased (simple one-choice question to choose one option: decreased, remained stable, or increased). At the same time, the question about recognizable body mass changes during the COVID-19 pandemic was supported by the information about the current height and weight of participants, as well as their height and weight before the period of remote learning. The participants were asked about their current weight, and afterwards they were asked to within a simple one-choice question choose one option: that it decreased, remained stable, or increased during the COVID-19 pandemic. If they declared that their weight increased or decreased, they were asked to declare what was their weight before the COVID-19 pandemic. The Body Mass Index (BMI) values were calculated for each participant using a standard Quetelet equation [51] and they were assessed using the Polish growth reference values, based on gender and age [52], with dedicated software [53]. As for physical activity, respondents were classified as those whose body mass decreased, remained stable, or increased during the COVID-19 pandemic. In order to obtain reliable answers, respondents were informed that their answers will not be judged in any way and that they are needed only for the scientific research.

### 2.4. Statistical Analysis

The statistical analysis was based on the comparison of the frequency of the specific answers provided by respondents for the periods before and during the COVID-19 pandemic, assessed based on identical questions formulated twice (separately for the indicated periods), while the AFHC was used. The assessment included assessment of the following factors:

− Period of the COVID-19 pandemic (comparison of the answers provided for the period before and during the COVID-19 pandemic);
− Physical activity changes (comparison of the answers provided by respondents declaring that their physical activity decreased, remained stable, or increased during the COVID-19 pandemic);
− Body mass changes (comparison of the answers provided by respondents declaring that their body mass decreased, remained stable, or increased during the COVID-19 pandemic).

The statistical analysis was conducted while using the chi-square test (for the comparison of the frequency of specific answers for the period before and during the COVID-19 pandemic) and McNemar’s test (for the assessment of changes of answers provided by specific respondents in longitudinal analysis, while comparing period before and during the COVID-19 pandemic). The Statgraphics Plus for Windows 5.1 software (Statgraphics Technologies Inc., The Plains, VA, USA) was used. The values of *p* ≤ 0.05 were interpreted as statistically significant differences.

## 3. Results

### 3.1. Comparison of Results before and during the COVID-19 Pandemic

The food purchase habits assessed while using AFHC in the period before the COVID-19 pandemic and during the COVID-19 pandemic, as declared by the adolescents from the DAY-19 Study cohort, are presented in Table 1. During the COVID-19 pandemic, the number of respondents who declared choosing a low-fat lunch away from home decreased (40.4% vs. 34.0%, *p* < 0.0001). At the same time, the number of respondents who declared often buying pastries or cakes decreased (33.6% vs. 24.1%, *p* < 0.0001), but the number of respondents who declared buying a low-fat crisps brand increased (22.6% vs. 24.8%, *p* < 0.05). The longitudinal analysis confirmed the significant changes of the food purchase habits in the period of the COVID-19 pandemic, which was stated for all the habits except for choosing a low-fat option while having lunch away from home. 

The food preparation habits assessed while using AFHC in the period before the COVID-19 pandemic and during the COVID-19 pandemic, as declared by the adolescents from the DAY-19 Study cohort, are presented in Table 2. During the COVID-19 pandemic, the number of respondents who declared trying to keep overall fat intake down increased (54.3% vs. 60.0%, *p* < 0.01) and the number of respondents who declared trying to keep overall sugar intake down also increased (55.6% vs. 61.7%, *p* < 0.01). At the same time, the number of respondents who declared eating at least one serving of vegetables or salad with evening meal increased (75.5% vs. 79.8%, *p* < 0.01), while the number of respondents who declared usually including some chocolate and/or biscuits in a packed lunch decreased (32.6% vs. 23.1%, *p* < 0.0001). The longitudinal analysis confirmed the significant changes of the food preparation habits in the period of the COVID-19 pandemic, which was stated for all the habits except for eating at least one serving of vegetables with the evening meal.

The food consumption habits assessed while using AFHC in the period before the COVID-19 pandemic and during the COVID-19 pandemic, as declared by the adolescents from the DAY-19 Study cohort, are presented in Table 3. During the COVID-19 pandemic, the number of respondents who declared making sure that they eat at least one serving of fruit a day increased (82.3% vs. 86.3%, *p* < 0.004) and eating at least three servings of fruit most days increased (47.4% vs. 56.6%, *p* < 0.0001), while the number of respondents who declared making sure that they eat at least one serving of vegetables or salad a day also increased (71.2% vs. 78.4%, *p* < 0.0001). Moreover, the number of respondents who declared trying to ensure that they eat plenty of fruit and vegetables increased (75.5% vs. 82.2%, *p* < 0.0001), while the number of respondents who declared often choosing a fruit when they have a snack between meals increased (45.9% vs. 51.4%, *p* < 0.05) and the number of respondents who declared eating at least three servings of fruit most days also increased (47.4% vs. 56.6%, *p* < 0.0001). At the same time, the number of respondents who declared that they generally try to have a healthy diet also increased (72.2% vs. 80.1%, *p* < 0.0001). The longitudinal analysis confirmed significant changes of food consumption habits in the period of the COVID-19 pandemic, which was stated for all the habits except for eating sweet snacks between meals.

### 3.2. Comparison of Results Associated with Body Mass Changes

The food purchase habits assessed while using AFHC in the period during the COVID-19 pandemic, as declared by the adolescents from the DAY-19 Study cohort in sub-groups of adolescents declaring decreased, stable, and increased body mass during the COVID-19 pandemic are presented in Table 4. A lower number of respondents who reported increased body mass, when compared with those who reported decreased body mass, declared often choosing a low-fat option while having lunch away from home (28.3% vs. 46.1%, *p* < 0.0001), rarely eating takeaway meals (80.1% vs. 86.8%, *p* < 0.05), choosing a low-fat crisps brand (23.9% vs. 28.8%, *p* < 0.001), and choosing a diet soft drink (34.9% vs. 58.0%, *p* < 0.0001), as well as usually choosing the healthiest dessert or pudding in a restaurant (15.8% vs. 25.5%, *p* < 0001). At the same time, a higher number of respondents who reported increased body mass, when compared with those who reported decreased body mass, declared often buying pastries or cakes (28.3% vs. 20.2%, *p* < 0.05).

The food preparation habits assessed while using AFHC in the period during the COVID-19 pandemic, as declared by the adolescents from the DAY-19 Study cohort, in sub-groups of adolescents declaring decreased, stable, and increased body mass during the COVID-19 pandemic are presented in Table 5. A lower number of respondents who reported increased body mass, when compared with those who reported decreased body mass, declared avoiding eating fried foods (34.9% vs. 54.7%, *p* < 0.0001), trying to keep their overall fat intake down (53.7% vs. 72.8%, *p* < 0.0001), trying to keep their overall sugar intake down (57.6% vs. 73.7%, *p* < 0.0001), trying to have something low in fat while having a dessert at home (32.5% vs. 43.2%, *p* < 0.0001), usually eating at least one serving of vegetables or salad with evening meal (76.6% vs. 83.5%, *p* < 0.05), and usually including some chocolate and/or biscuits while having a packed lunch (26.7% vs. 46.1%, *p* < 0.0001).

The food consumption habits assessed while using AFHC in the period during the COVID-19 pandemic, as declared by the adolescents from the DAY-19 Study cohort, in sub-groups of adolescents declaring decreased, stable, and increased body mass during the COVID-19 pandemic are presented in Table 6. A higher number of respondents who reported increased body mass, when compared with those who reported decreased body mass, declared usually eating a dessert or pudding if there is one available (73.8% vs. 64.2%, *p* < 0.01) and often eating sweet snacks between meals (55.6% vs. 32.1%, *p* < 0.0001). At the same time, lower number of respondents who reported increased body mass, when compared with those who reported decreased body mass, declared avoiding eating lots of sausages and burgers (57.8% vs. 65.0%, *p* < 0.001), making sure they eat at least one serving of vegetables or salad a day (74.9% vs. 85.6%, *p* < 0.01), trying to ensure they eat plenty of fruit and vegetables (78.6% vs. 86.0%, *p* < 0.05), often choosing fruit while having a snack between meals (48.9% vs. 56.4%, *p* < 0.0001), eating at least three servings of fruit most days (53.3% vs. 69.1%, *p* < 0.0001), and generally trying to have a healthy diet.

### 3.3. Comparison of Results Associated with Physcial Activity Changes

The food purchase habits assessed while using AFHC in the period during the COVID-19 pandemic, as declared by the adolescents from the DAY-19 Study cohort, in sub-groups of adolescents declaring decreased, stable, and increased physical activity during the COVID-19 pandemic are presented in Table 7. A lower number of respondents who reported decreased physical activity, when compared with those who reported increased physical activity, declared often choosing a low-fat option while having lunch away from home (27.9% vs. 38.8%, *p* < 0.01), often choosing a low-fat crisps brand (20.8% vs. 25.5%, *p* < 0.001), usually choosing a diet soft drink (28.2% vs. 48.2%, *p* < 0.0001), and usually choosing the healthiest dessert or pudding in a restaurant (29.7% vs. 37.2%, *p* < 0.0001). At the same time, a higher number of respondents who reported decreased physical activity, when compared with those who reported increased physical activity, declared often buying pastries or cakes (27.1% vs. 25.2%, *p* < 0.05).

The food preparation habits assessed while using AFHC in the period during the COVID-19 pandemic, as declared by the adolescents from the DAY-19 Study cohort, in sub-groups of adolescents declaring decreased, stable, and increased physical activity during the COVID-19 pandemic are presented in Table 8. A lower number of respondents who reported decreased physical activity, when compared with those who reported increased physical activity, declared usually avoiding eating fried foods (32.4% vs. 46.3%, *p* < 0.0001), trying to keep their overall fat intake down (48.9% vs. 65.7%, *p* < 0.0001), trying to keep their overall sugar intake down (50.8% vs. 68.5%, *p* < 0.0001), trying to have something low in fat while having a dessert at home (29.6% vs. 37.2%, *p* < 0.0001), and usually eating at least one serving of vegetables or salad with evening meal (75.8% vs. 84.6%, *p* < 0.01). At the same time, a higher number of respondents who reported decreased physical activity, when compared with those who reported increased physical activity, declared usually spreading butter or margarine on bread thinly (69.3% vs. 65.1%, *p* < 0.05), and usually including some chocolate and/or biscuits to a packed lunch (27.3% vs. 18.6%, *p* < 0.05).

The food consumption habits assessed while using AFHC in the period during the COVID-19 pandemic, as declared by the adolescents from the DAY-19 Study cohort, in sub-groups of adolescents declaring decreased, stable, and increased physical activity during the COVID-19 pandemic are presented in Table 9. A higher number of respondents who reported decreased physical activity, when compared with those who reported increased physical activity, declared usually eating a dessert or pudding if there is one available (79.0% vs. 70.4%, *p* < 0.0001), and often eating sweet snacks between meals (55.8% vs. 43.0%, *p* < 0.0001). At the same time, a lower number of respondents who reported decreased physical activity, when compared with those who reported increased physical activity, declared making sure they eat at least one serving of fruit a day (80.5% vs. 89.5%, *p* < 0.0001), avoiding eating lots of sausages and burgers (58.8% vs. 61.7%, *p* < 0.001), making sure they eat at least one serving of vegetables or salad a day (71.6% vs. 82.4%, *p* < 0.0001), trying to ensure they eat plenty of fruit and vegetables (73.3% vs. 87.4%, *p* < 0.0001), often choosing fruit while having a snack between meals (41.0% vs. 59.3%, *p* < 0.0001), eating at least three servings of fruit most days (45.8% vs. 66.2%, *p* < 0.0001), and generally trying to have a healthy diet (70.8% vs. 84.6%, *p* < 0.0001).

### 3.4. Analysis of the Interfering Factors

The food purchase habits assessed while using AFHC in the period before the COVID-19 pandemic (Supplementary Table S1) and the period during the COVID-19 pandemic (Supplementary Table S2), the food preparation habits assessed in the period before the COVID-19 pandemic (Supplementary Table S3) and the period during the COVID-19 pandemic (Supplementary Table S4), as well as the food consumption habits assessed in the period before the COVID-19 pandemic (Supplementary Table S5) and the period during the COVID-19 pandemic (Supplementary Table S6), stratified by gender, indicated some differences. However, for the majority of habits no gender-dependent differences were stated.

The food purchase habits assessed while using AFHC in the period before the COVID-19 pandemic (Supplementary Table S7) and the period during the COVID-19 pandemic (Supplementary Table S8), the food preparation habits assessed in the period before the COVID-19 pandemic (Supplementary Table S9) and the period during the COVID-19 pandemic (Supplementary Table S10), as well as the food consumption habits assessed in the period before the COVID-19 pandemic (Supplementary Table S11) and the period during the COVID-19 pandemic (Supplementary Table S12), stratified by urban/rural environment, indicated some differences. However, for the majority of habits no environment-dependent differences were stated.

## 4. Discussion

Social isolation, caused by the COVID-19 pandemic, induced a lot of changes of everyday life, influencing also the nutrition of children and adolescents. However, based on a review by Stavridou et al. [54], it may be stated that the majority of studies indicated negative changes into less beneficial dietary behaviors. At the same time, in the presented study, it was observed that the majority of changes of food habits in the studied group were positive, as they were associated with reduced fat, sugar, and sweets intake, accompanied by increased consumption of fruit and vegetables. Similarly, in the previous study conducted in Poland, in a population of older adolescents [41], it was noted that during the COVID-19 pandemic, when compared with the period before, a lower number of respondents reported buying pastries, cakes or crisps, and eating a dessert or pudding, as well as a higher number of respondents reported avoiding fried foods, trying to keep their overall sugar intake down, and eating at least one serving of vegetables or salad a day and at least three servings of fruit most days. However, authors of the referred study indicated that similar share of their respondents changed their food habits into more beneficial as into less beneficial ones, and that it was probable that beneficial food habits were forced by the lockdown and the resultant limited possibilities to consume meals in restaurants [41]. Moreover, a recent Polish study revealed also the problem associated with potentially reduced frequency of using catering facilities, influenced not by a need for following a healthy diet, but by the fear of getting sick with COVID-19 [55].

The results of numerous studies indicate a growing problem of the excessive body mass of adolescents during the COVID-19 pandemic [26,54,56], so not only the problem of changing food habits, but also their association with changes of body mass should be reflected. In the presented study, adolescents declaring increase of their body mass during the COVID-19 pandemic were characterized by worse food habits than those who did not declare it. However, especially alarming is that social isolation during this period not only may have induced development of overweight and obesity, but also may have intensified the problem in case of adolescents already being overweight or obese before the pandemic [57]. Some authors indicate that following dietary recommendations during the lockdown was especially hard for excessive body mass children, due to their increase in sedentary lifestyle and increase in feeling hungry [22].

Among various factors influencing food habits of adolescents, there is an important impact of their parents on the food choices of their progeny [58]. In spite of the fact that it was not assessed within the presented study, the family habits may significantly influence the analyzed population, so it must be emphasized that the COVID-19 pandemic may have a direct influence on food habits of children and an indirect influence (it may influence habits of parents, and they may consequently influence habits of their progeny). The results of the study by Larson et al. [59] revealed that consuming family dishes may exert a positive influence on the quality of diet and dietary patterns in young adults. This is also confirmed by the results of a study by Merten et al. [60], as they noted that for breakfast consumption, an important determinant is the presence of a parent while consuming this meal. In addition, during the COVID-19 pandemic some researchers noted positive changes of nutrition of families, as they spent more time preparing dishes together [23,24]. On the other hand, some authors revealed also negative changes of the nutritional behaviors within families during the COVID-19 pandemic, which were noted especially in the initial phase of the pandemic [61]. In spite of the fact that the influence of family members was not studied in the DAY-19 Study, it cannot be ruled out, taking into account that family members influence one another their food habits, so the negative food habits of adolescents may be shared by all their family members. At the same time, it must also be emphasized that the excessive body mass of children may be associated with excessive body mass of their parents, which may be supposed to be the other serious problem in the period of the COVID-19 pandemic.

The previous studies by other authors indicated that social isolation and prolonging stays at home, commonly associated with internet and social media overuse, are a factors which may have a negative impact on a mental health of children and adolescents in this period, causing anxiety symptoms, depression, and post-traumatic stress [62]. Some studies indicate that both heathy diet [63] and physical activity may positively influence the mental health of children [64,65]. The studies conducted in Great Britain, during the COVID-19 pandemic revealed that a higher level of physical activity may counteract the negative effects of COVID-19 fears on mental health and well-being [66]. As in the presented study, the increased physical activity was associated with better food habits, both of them combined may be especially valuable. Moreover, a higher level of physical activity is commonly associated with improved dietary behaviors [67], as it was stated within the DAY-19 Study especially for dietary behaviors associated with fruit and vegetables’ consumption. At the same time, Cavadini et al. [33] also noted that adolescents characterized by a higher level of physical activity simultaneously presented a higher frequency of consuming fruit, fruit juices, and salads than in the case of other adolescents.

It should be indicated that increased screen time, which is commonly observed during the COVID-19 pandemic [68], may also be an important factor influencing food habits. A study conducted in a Spanish population of children and adolescents during the COVID-19 pandemic revealed that increased screen time was associated with worse dietary behaviors, including lower intake of fruit, vegetables, fish, pulses, and nuts, but higher intake of fast foods and sweets [69]. Similar observations of increased screen time have also been indicated during the COVID-19 pandemic in the population of Polish adolescents [2]. Taking this into account, it may be supposed that changing food habits which were noted in the conducted study may be also associated with increased screen time and mediated by snacking behaviors [70].

Taking into account the fact that during the period of adolescence some physical activity patterns are developed and strengthened, the observations made in this group during the COVID-19 pandemic may be especially important. The research conducted before the pandemic revealed that adolescents declaring a higher physical activity level at the same time have better nutritional behaviors than the other adolescents [29]. Moreover, during the COVID-19 pandemic the physical activity level in this group has significantly decreased, associated with lack of physical education lessons at school, but also with closed playgrounds and courts [28,71,72] being indicated by adolescents as a reason of their reduced physical activity [27]. This problem may have existed in the studied population and may have caused that only adolescents of the highest motivation had an adequate physical activity level, as they were seeking alternative options to practicing sports.

As indicated by Barwais [73], during the COVID-19 pandemic physical activity levels decreased, and this was stated for the activities practiced alone, with friends, families, and team sports. Interestingly, a study conducted by Kaur et al. [74] in a population of adults revealed that, especially in the initial phase of the pandemic and resultant lockdowns, respondents had low motivation to practice any sports, while afterwards the situation changed and their motivation increased, as similarly observed in another study for following a healthy diet [61]. This confirms that even during the pandemic, there are possibilities to increase physical activity and to correct improper dietary behaviors, but dedicated tools and adequate time should be applied. At the same time, any digital platforms may be indicated as a promising options, as using them is associated with a higher level of general physical activity during the COVID-19 pandemic and it may motivate users to practice any sport and to develop an individual physical activity routine [75]. 

In spite of the fact that the conducted study presented some novel observations of the food habits during the COVID-19 pandemic and their association with physical activity and body mass changes in a Polish population of primary school adolescents, some limitations of the study should be listed. The most important issue is associated with the fact that participants were asked about their situation before the COVID-19 pandemic, so gathering retrospective data was included, which may generate so-called recall bias, being a systematic error caused by possible errors of the recollections retrieved by study participants regarding events or experiences from the past. However, it should be indicated that the COVID-19 pandemic was unexpected, so it was not possible to plan an adequate prospective study to conduct such an assessment without recall bias. As such, in spite of the existing bias, the study provided some valuable observations. At the same time, there is also a risk of a self-selection bias, which may have caused that some potential participants of the study did not intend to participate and those who intended had some specific characteristics. The other issue is associated with a fact that participants were not asked about their diet-related diseases diagnosed during the COVID-19 pandemic, and about those diagnosed previously, so some of the observed results may be influenced by specific dietary recommendations which must be followed due to some diseases or disorders. Last but not least, in spite of the fact that some interfering factors were taken into account, there are also other potential factors that were not studied, such as changes of the family socioeconomic status, which may have been caused by the pandemic (as a result of, e.g., unemployment) and also may have influenced the food habits of adolescents. 

## 5. Conclusions

In the period of the COVID-19 pandemic and resultant remote education, adolescents in Poland presented different food habits than before. Positive food purchase, preparation, and consumption habits were observed mainly in the sub-groups of adolescents declaring decreased body mass or increased physical activity during the COVID-19 pandemic. It may be suggested that physical activity may support positive changes of dietary behaviors and, when combined positive changes of diet and increased physical activity, they may effectively promote body mass reduction.

## Figures and Tables

**Table 1:** The food purchase habits assessed while using Adolescents’ Food Habits Checklist (AFHC) in the period before the COVID-19 pandemic and during the COVID-19 pandemic, as declared by the adolescents from the Diet and Activity of Youth during COVID-19 (DAY-19) Study cohort (*n* = 1333).

Food Purchase Habits Assessed within AFHC *		Before COVID-19 Pandemic	During the COVID-19 Pandemic	p **	p ***
If I am having lunch away from home, I often choose a low-fat option	True	538 (40.4%)	453 (34.0%)	<0.0001	0.7344
	False	392 (29.4%)	365 (27.4%)		
	Not applicable	403 (30.2%)	515 (38.6%)		
If I am buying crisps, I often choose a low-fat brand	True	301 (22.6%)	331 (24.8%)	0.0139	<0.0001
	False	799 (59.9%)	726 (54.5%)		
	Not applicable	233 (17.5%)	276 (20.7%)		
I often buy pastries or cakes	True	448 (33.6%)	322 (24.1%)	<0.0001	<0.0001
	False	885 (66.4%)	1011 (75.8%)		
I rarely eat takeaway meals	True	1085 (81.4%)	1108 (83.1%)	0.2435	<0.0001
	False	248 (18.6%)	225 (16.9%)		
When I am buying a soft drink, I usually choose a diet drink	True	481 (36.1%)	530 (39.8%)	0.0505	0.0001
	False	852 (63.9%)	803 (60.2%)		
If I am having a dessert or pudding in a restaurant, I usually choose the healthiest one	True	214 (16.5%)	226 (16.9%)	0.2732	<0.0001
	False	576 (43.2%)	535 (40.2%)		
	Not applicable	543 (40.7%)	572 (42.9%)		

* AFHC–Adolescents’ Food Habits Checklist [37]; ** chi-square test (for the comparison of the frequency of specific answers); *** McNemar’s test (for longitudinal analysis).

**Table 2:** The food preparation habits assessed while using Adolescents’ Food Habits Checklist (AFHC) in the period before the COVID-19 pandemic and during the COVID-19 pandemic, as declared by the adolescents from the Diet and Activity of Youth during COVID-19 (DAY-19) Study cohort (*n* = 1333).

Food Preparation Habits Assessed within AFHC *		Before COVID-19 Pandemic	During COVID-19 Pandemic	p **	p ***
I usually avoid eating fried foods	True	509 (38.2%)	536 (40.2%)	0.2842	<0.0001
	False	824 (61.8%)	797 (59.8%)		
I try to keep my overall fat intake down	True	724 (54.3%)	800 (60.0%)	0.0029	<0.0001
	False	609 (45.7%)	533 (40.0%)		
I try to keep my overall sugar intake down	True	741 (55.6%)	822 (61.7%)	0.0014	<0.0001
	False	592 (44.4%)	511 (38.3%)		
If I am having a dessert at home, I try to have something low in fat	True	433 (32.5%)	483 (36.2%)	0.1249	<0.0001
	False	679 (50.9%)	642 (48.2%)		
	Not applicable	221 (16.6%)	208 (15.6%)		
I usually eat at least one serving of vegetables (excluding potatoes) or salad with my evening meal	True	1006 (75.5%)	1064 (79.8%)	0.0070	<0.0001
	False	327 (24.5%)	269 (20.2%)		
When I put butter or margarine on bread, I usually spread it thinly	True	916 (68.7%)	912 (68.4%)	0.7811	0.7604
	False	198 (14.9%)	190 (14.3%)		
	Not applicable	219 (16.4%)	231 (17.3%)		
If I have a packed lunch, I usually include some chocolate and/or biscuits	True	435 (32.6%)	308 (23.1%)	<0.0001	<0.0001
	False	751 (56.3%)	795 (59.6%)		
	Not applicable	147 (11.1%)	230 (17.3%)		
I often have cream on desserts	True	288 (21.6%)	291 (21.8%)	0.7115	<0.0001
	False	886 (66.5%)	870 (65.3%)		
	Not applicable	159 (11.9%)	172 (12.9%)		

* AFHC–Adolescents’ Food Habits Checklist [37]; ** chi-square test (for the comparison of the frequency of specific answers); *** McNemar’s test (for longitudinal analysis).

**Table 3:** The food consumption habits assessed while using Adolescents’ Food Habits Checklist (AFHC) in the period before the COVID-19 pandemic and during the COVID-19 pandemic, as declared by the adolescents from the Diet and Activity of Youth during COVID-19 (DAY-19) Study cohort (*n* = 1333).

Food Consumption Habits Assessed within AFHC *		Before COVID-19 Pandemic	During COVID-19 Pandemic	p **	p ***
I usually eat a dessert or pudding if there is one available	True	953 (71.5%)	963 (72.2%)	0.6663	<0.0001
	False	380 (28.5%)	370 (27.8%)		
I make sure I eat at least one serving of fruit a day	True	1097 (82.3%)	1151 (86.3%)	0.0040	<0.0001
	False	236 (17.7%)	182 (13.7%)		
I avoid eating lots of sausages and burgers	True	783 (58.8%)	809 (60.7%)	0.4219	<0.0001
	False	343 (25.7%)	314 (23.5%)		
	Not applicable	207 (15.5%)	210 (15.8%)		
I make sure I eat at least one serving of vegetables or salad a day	True	949 (71.2%)	1045 (78.4%)	<0.0001	<0.0001
	False	384 (28.8%)	288 (21.6%)		
I try to ensure I eat plenty of fruit and vegetables	True	1009 (75.7%)	1096 (82.2%)	<0.0001	<0.0001
	False	324 (24.3%)	237 (17.8%)		
I often eat sweet snacks between meals	True	623 (46.7%)	641 (48.1%)	0.4853	0.2954
	False	710 (53.3%)	692 (51.9%)		
When I have a snack between meals, I often choose fruit	True	611 (45.9%)	686 (51.4%)	0.0111	<0.0001
	False	558 (41.8%)	489 (36.7%)		
	Not applicable	164 (12.3%)	158 (11.9%)		
I eat at least three servings of fruit most days	True	632 (47.4%)	754 (56.6%)	<0.0001	<0.0001
	False	701 (52.6%)	579 (43.4%)		
I generally try to have a healthy diet	True	963 (72.2%)	1068 (80.1%)	<0.0001	<0.0001
	False	370 (27.8%)	265 (19.9%)		

* AFHC–Adolescents’ Food Habits Checklist [37]; ** chi-square test (for the comparison of the frequency of specific answers); *** McNemar’s test (for longitudinal analysis).

**Table 4:** The food purchase habits assessed while using Adolescents’ Food Habits Checklist (AFHC) in the period during the COVID-19 pandemic, as declared by the adolescents from the Diet and Activity of Youth during COVID-19 (DAY-19) Study cohort (*n* = 1333) in sub-groups of adolescents declaring decreased, stable, and increased body mass during the COVID-19 pandemic.

Food Purchase Habits Assessed within AFHC *		Decreased Body Mass during COVID-19 Pandemic (*n* = 243)	Stable Body Mass during COVID-19 Pandemic (*n* = 552)	Increased Body Mass during COVID-19 Pandemic (*n* = 538)	*p* **
If I am having lunch away from home, I often choose a low-fat option	True	112 (46.1%)	189 (34.3%)	152 (28.3%)	<0.0001
	False	49 (20.2%)	149 (26.9%)	167 (31.0%)	
	Not applicable	82 (33.7%)	214 (38.8%)	219 (40.7%)	
If I am buying crisps, I often choose a low-fat brand	True	70 (28.8%)	132 (23.9%)	129 (23.9%)	0.0004
	False	106 (43.6%)	299 (54.2%)	321 (59.7%)	
	Not applicable	67 (27.6%)	121 (21.9%)	88 (16.4%)	
I often buy pastries or cakes	True	49 (20.2%)	121 (21.9%)	152 (28.3%)	0.0139
	False	194 (79.8%)	431 (78.1%)	386 (71.7%)	
I rarely eat takeaway meals	True	211 (86.8%)	466 (84.4%)	431 (80.1%)	0.0384
	False	32 (13.2%)	86 (15.6%)	107 (19.9%)	
When I am buying a soft drink, I usually choose a diet drink	True	141 (58.0%)	201 (36.4%)	188 (34.9%)	<0.0001
	False	102 (42.0%)	351 (63.6%)	350 (65.1%)	
If I am having a dessert or pudding in a restaurant, I usually choose the healthiest one	True	62 (25.5%)	79 (14.3%)	85 (15.8%)	<0.0001
	False	61 (25.1%)	235 (42.6%)	239 (44.4%)	
	Not applicable	120 (49.4%)	238 (43.1%)	214 (39.8%)	

* AFHC–Adolescents’ Food Habits Checklist [37]; ** chi-square test.

**Table 5:** The food preparation habits assessed while using Adolescents’ Food Habits Checklist (AFHC) in the period during the COVID-19 pandemic, as declared by the adolescents from the Diet and Activity of Youth during COVID-19 (DAY-19) Study cohort (*n* = 1333) in sub-groups of adolescents declaring decreased, stable, and increased body mass during the COVID-19 pandemic.

Food Preparation Habits Assessed within AFHC *		Decreased Body Mass during COVID-19 Pandemic (*n* = 243)	Stable Body Mass during COVID-19 Pandemic (*n* = 552)	Increased Body Mass during COVID-19 Pandemic (*n* = 538)	*p* **
I usually avoid eating fried foods	True	133 (54.7%)	215 (38.9%)	188 (34.9%)	<0.0001
	False	110 (45.3%)	337 (61.1%)	350 (65.1%)	
I try to keep my overall fat intake down	True	177 (72.8%)	334 (60.5%)	289 (53.7%)	<0.0001
	False	66 (27.2%)	218 (39.5%)	249 (46.3%)	
I try to keep my overall sugar intake down	True	179 (73.7%)	333 (60.3%)	310 (57.6%)	<0.0001
	False	64 (26.3%)	219 (39.7%)	228 (42.4%)	
If I am having a dessert at home, I try to have something low in fat	True	105 (43.2%)	203 (36.8%)	175 (32.5%)	<0.0001
	False	82 (33.8%)	270 (48.9%)	290 (53.9%)	
	Not applicable	56 (23.0%)	79 (14.3%)	73 (13.6%)	
I usually eat at least one serving of vegetables (excluding potatoes) or salad with my evening meal	True	203 (83.5%)	449 (81.3%)	412 (76.6%)	0.0411
	False	40 (16.5%)	103 (18.7%)	126 (23.4%)	
When I put butter or margarine on bread, I usually spread it thinly	True	158 (65.0%)	379 (68.6%)	375 (69.7%)	0.4036
	False	35 (14.4%)	74 (13.5%)	81 (15.1%)	
	Not applicable	50 (20.6%)	99 (17.9%)	82 (15.2%)	
If I have a packed lunch, I usually include some chocolate and/or biscuits	True	112 (46.1%)	121 (21.9%)	144 (26.7%)	<0.0001
	False	49 (20.2%)	339 (61.4%)	299 (55.6%)	
	Not applicable	82 (33.7%)	92 (16.7%)	95 (17.7%)	
I often have cream on desserts	True	49 (20.2%)	110 (19.9%)	132 (24.5%)	0.2642
	False	157 (64.6%)	374 (67.8%)	339 (63.0%)	
	Not applicable	37 (15.2%)	68 (12.3%)	67 (12.5%)	

* AFHC–Adolescents’ Food Habits Checklist [37]; ** chi-square test.

**Table 6:** The food consumption habits assessed while using Adolescents’ Food Habits Checklist (AFHC) in the period during the COVID-19 pandemic, as declared by the adolescents from the Diet and Activity of Youth during COVID-19 (DAY-19) Study cohort (*n* = 1333) in sub-groups of adolescents declaring decreased, stable, and increased body mass during the COVID-19 pandemic.

Food Consumption Habits Assessed within AFHC *		Decreased Body Mass during COVID-19 Pandemic (*n* = 243)	Stable Body Mass during COVID-19 Pandemic (*n* = 552)	Increased Body Mass during COVID-19 Pandemic (*n* = 538)	p **
I usually eat a dessert or pudding if there is one available	True	156 (64.2%)	410 (74.3%)	397 (73.8%)	0.0081
	False	87 (35.8%)	142 (25.7%)	141 (26.2%)	
I make sure I eat at least one serving of fruit a day	True	212 (87.3%)	482 (87.3%)	457 (84.9%)	0.4709
	False	31 (12.7%)	70 (12.7%)	81 (15.1%)	
I avoid eating lots of sausages and burgers	True	158 (65.0%)	340 (61.6%)	311 (57.8%)	<0.0001
	False	32 (13.2%)	132 (23.9%)	150 (27.9%)	
	Not applicable	53 (21.8%)	80 (14.5%)	77 (14.3%)	
I make sure I eat at least one serving of vegetables or salad a day	True	208 (85.6%)	434 (78.6%)	403 (74.9%)	0.0035
	False	35 (14.4%)	118 (21.4%)	135 (25.1%)	
I try to ensure I eat plenty of fruit and vegetables	True	209 (86.0%)	464 (84.1%)	423 (78.6%)	0.0148
	False	34 (14.0%)	88 (15.9%)	115 (21.4%)	
I often eat sweet snacks between meals	True	78 (32.1%)	264 (47.8%)	299 (55.6%)	<0.0001
	False	165 (67.9%)	288 (52.2%)	239 (44.4%)	
When I have a snack between meals, I often choose fruit	True	137 (56.4%)	286 (51.8%)	263 (48.9%)	<0.0001
	False	60 (24.7%)	207 (37.5%)	222 (41.3%)	
	Not applicable	46 (18.9%)	59 (10.7%)	53 (9.8%)	
I eat at least three servings of fruit most days	True	168 (69.1%)	299 (54.2%)	287 (53.3%)	<0.0001
	False	75 (30.9%)	253 (45.8%)	251 (46.7%)	
I generally try to have a healthy diet	True	219 (90.1%)	446 (80.8%)	403 (74.9%)	<0.0001
	False	24 (9.9%)	106 (19.2%)	135 (25.1%)	

* AFHC–Adolescents’ Food Habits Checklist [37]; ** chi-square test.

**Table 7:** The food purchase habits assessed while using Adolescents’ Food Habits Checklist (AFHC) in the period during the COVID-19 pandemic, as declared by the adolescents from the Diet and Activity of Youth during COVID-19 (DAY-19) Study cohort (*n* = 1333) in sub-groups of adolescents declaring decreased, stable, and increased physical activity during the COVID-19 pandemic.

Food Purchase Habits Assessed within AFHC *		Decreased Physical Activity during COVID-19 Pandemic (*n* = 476)	Stable Physical Activity during COVID-19 Pandemic (*n* = 390)	Increased Physical Activity during COVID-19 Pandemic (*n* = 467)	*p* **
If I am having lunch away from home, I often choose a low-fat option	True	133 (27.9%)	139 (35.6%)	181 (38.8%)	0.0020
	False	156 (32.8%)	96 (24.6%)	113 (24.2%)	
	Not applicable	187 (39.4%)	155 (39.8%)	173 (37.0%)	
If I am buying crisps, I often choose a low-fat brand	True	99 (20.8%)	113 (28.9%)	119 (25.5%)	<0.0001
	False	309 (64.9%)	193 (49.6%)	224 (47.9%)	
	Not applicable	68 (14.3%)	84 (21.5%)	124 (26.6%)	
I often buy pastries or cakes	True	129 (27.1%)	75 (19.2%)	118 (25.3%)	0.0209
	False	347 (72.9%)	315 (80.8%)	349 (74.7%)	
I rarely eat takeaway meals	True	398 (83.6%)	333 (85.4%)	377 (80.7%)	0.1815
	False	78 (16.4%)	57 (14.6%)	90 (19.3%)	
When I am buying a soft drink, I usually choose a diet drink	True	134 (28.2%)	171 (43.8%)	225 (48.2%)	<0.0001
	False	342 (71.8%)	219 (56.2%)	242 (51.8%)	
If I am having a dessert or pudding in a restaurant, I usually choose the healthiest one	True	141 (29.7%)	168 (43.1%)	174 (37.2%)	<0.0001
	False	278 (58.4%)	161 (41.3%)	203 (43.5%)	
	Not applicable	57 (11.9%)	61 (15.6%)	90 (19.3%)	

* AFHC–Adolescents’ Food Habits Checklist [37]; ** chi-square test.

**Table 8:** The food preparation habits assessed while using Adolescents’ Food Habits Checklist (AFHC) in the period during the COVID-19 pandemic, as declared by the adolescents from the Diet and Activity of Youth during COVID-19 (DAY-19) Study cohort (*n* = 1333) in sub-groups of adolescents declaring decreased, stable, and increased physical activity during the COVID-19 pandemic.

Food Preparation Habits Assessed within AFHC *		Decreased Physical Activity during COVID-19 Pandemic (*n* = 476)	Stable Physical Activity during COVID-19 Pandemic (*n* = 390)	Increased Physical Activity during COVID-19 Pandemic (*n* = 467)	*p* **
I usually avoid eating fried foods	True	154 (32.4%)	166 (42.6%)	216 (46.3%)	<0.0001
	False	322 (67.6%)	224 (57.4%)	251 (53.7%)	
I try to keep my overall fat intake down	True	233 (48.9%)	260 (66.7%)	307 (65.7%)	<0.0001
	False	243 (51.1%)	130 (33.3%)	160 (34.2%)	
I try to keep my overall sugar intake down	True	242 (50.8%)	260 (66.7%)	320 (68.5%)	<0.0001
	False	234 (49.2%)	130 (33.3%)	147 (31.5%)	
If I am having a dessert at home, I try to have something low in fat	True	141(29.6%)	168 (43.1%)	174 (37.2%)	<0.0001
	False	278 (58.4%)	161 (41.3%)	203 (43.5%)	
	Not applicable	57 (12.0%)	61 (15.6%)	90 (19.3%)	
I usually eat at least one serving of vegetables (excluding potatoes) or salad with my evening meal	True	361 (75.8%)	308 (79.0%)	395 (84.6%)	0.0033
	False	115 (24.2%)	82 (21.0%)	72 (15.4%)	
When I put butter or margarine on bread, I usually spread it thinly	True	330 (69.3%)	278 (71.3%)	304 (65.1%)	0.0135
	False	77 (16.2%)	38 (9.7%)	75 (16.1%)	
	Not applicable	69 (14.5%)	74 (19.0%)	88 (18.8%)	
If I have a packed lunch, I usually include some chocolate and/or biscuits	True	130 (27.3%)	91 (23.3%)	87 (18.6%)	0.0374
	False	266 (55.9%)	232 (59.5%)	297 (63.6%)	
	Not applicable	80 (16.8%)	67 (17.2%)	83 (17.8%)	
I often have cream on desserts	True	115 (24.1%)	75 (19.2%)	101 (21.6%)	0.1744
	False	311 (65.4%)	262 (67.2%)	297 (63.6%)	
	Not applicable	50 (10.5%)	53 (13.6%)	69 (14.8%)	

* AFHC–Adolescents’ Food Habits Checklist [37]; ** chi-square test.

**Table 9:** The food consumption habits assessed while using Adolescents’ Food Habits Checklist (AFHC) in the period during the COVID-19 pandemic, as declared by the adolescents from the Diet and Activity of Youth during COVID-19 (DAY-19) Study cohort (*n* = 1333) in sub-groups of adolescents declaring decreased, stable, and increased physical activity during the COVID-19 pandemic.

Food Consumption Habits Assessed within AFHC *		Decreased Physical Activity during COVID-19 Pandemic (*n* = 476)	Stable Physical Activity during COVID-19 Pandemic (*n* = 390)	Increased Physical Activity during COVID-19 Pandemic (*n* = 467)	p **
I usually eat a dessert or pudding if there is one available	True	376 (79.0%)	258 (66.1%)	329 (70.4%)	<0.0001
	False	100 (21.0%)	132 (33.8%)	138 (29.6%)	
I make sure I eat at least one serving of fruit a day	True	383 (80.5%)	350 (89.7%)	418 (89.5%)	<0.0001
	False	93 (19.5%)	40 (10.3%)	49 (10.5%)	
I avoid eating lots of sausages and burgers	True	280 (58.8%)	241 (61.8%)	288 (61.7%)	<0.0001
	False	142 (29.8%)	72 (18.5%)	100 (21.4%)	
	Not applicable	54 (11.4%)	77 (19.7%)	79 (16.9%)	
I make sure I eat at least one serving of vegetables or salad a day	True	341 (71.6%)	319 (81.8%)	385 (82.4%)	<0.0001
	False	135 (28.4%)	71 (18.2%)	82 (17.6%)	
I try to ensure I eat plenty of fruit and vegetables	True	349 (73.3%)	339 (86.9%)	408 (87.4%)	<0.0001
	False	127 (26.7%)	51 (13.1%)	59 (12.6%)	
I often eat sweet snacks between meals	True	266 (55.8%)	174 (44.6%)	201 (43.0%)	<0.0001
	False	210 (44.2%)	216 (55.4%)	266 (57.0%)	
When I have a snack between meals, I often choose fruit	True	195 (41.0%)	214 (54.9%)	277 (59.3%)	<0.0001
	False	223 (46.8%)	131 (33.6%)	135 (28.9%)	
	Not applicable	58 (12.2%)	45 (11.5%)	55 (11.8%)	
I eat at least three servings of fruit most days	True	218 (45.8%)	227 (58.2%)	309 (66.2%)	<0.0001
	False	258 (54.2%)	163 (41.8%)	158 (33.8%)	
I generally try to have a healthy diet	True	337 (70.8%)	336 (86.2%)	395 (84.6%)	<0.0001
	False	139 (29.2%)	54 (13.8%)	72 (15.4%)	

* AFHC–Adolescents’ Food Habits Checklist [37]; ** chi-square test.

## Supplementary Materials

The following are available online at https://www.mdpi.com/article/10.3390/nu13113711/s1. Supplementary Table S1. The food purchase habits assessed while using Adolescents’ Food Habits Checklist (AFHC) in the period before the COVID-19 pandemic, as declared by the adolescents from the Diet and Activity of Youth during COVID-19 (DAY-19) Study cohort (*n* = 1333), stratified by gender. Supplementary Table S2. The food purchase habits assessed while using Adolescents’ Food Habits Checklist (AFHC) in the period during the COVID-19 pandemic, as declared by the adolescents from the Diet and Activity of Youth during COVID-19 (DAY-19) Study cohort (*n* = 1333), stratified by gender. Supplementary Table S3. The food preparation habits assessed while using Adolescents’ Food Habits Checklist (AFHC) in the period before the COVID-19 pandemic, as declared by the adolescents from the Diet and Activity of Youth during COVID-19 (DAY-19) Study cohort (*n* = 1333), stratified by gender. Supplementary Table S4. The food preparation habits assessed while using Adolescents’ Food Habits Checklist (AFHC) in the period during the COVID-19 pandemic, as declared by the adolescents from the Diet and Activity of Youth during COVID-19 (DAY-19) Study cohort (*n* = 1333), stratified by gender. Supplementary Table S5. The food consumption habits assessed while using Adolescents’ Food Habits Checklist (AFHC) in the period before the COVID-19 pandemic, as declared by the adolescents from the Diet and Activity of Youth during COVID-19 (DAY-19) Study cohort (*n* = 1333), stratified by gender. Supplementary Table S6. The food consumption habits assessed while using Adolescents’ Food Habits Checklist (AFHC) in the period during the COVID-19 pandemic, as declared by the adolescents from the Diet and Activity of Youth during COVID-19 (DAY-19) Study cohort (*n* = 1333), stratified by gender. Supplementary Table S7. The food purchase habits assessed while using Adolescents’ Food Habits Checklist (AFHC) in the period before the COVID-19 pandemic, as declared by the adolescents from the Diet and Activity of Youth during COVID-19 (DAY-19) Study cohort (*n* = 1333), stratified by urban/rural environment. Supplementary Table S8. The food purchase habits assessed while using Adolescents’ Food Habits Checklist (AFHC) in the period during the COVID-19 pandemic, as declared by the adolescents from the Diet and Activity of Youth during COVID-19 (DAY-19) Study cohort (*n* = 1333), stratified by urban/rural environment. Supplementary Table S9. The food preparation habits assessed while using Adolescents’ Food Habits Checklist (AFHC) in the period before the COVID-19 pandemic, as declared by the adolescents from the Diet and Activity of Youth during COVID-19 (DAY-19) Study cohort (*n* = 1333), stratified by urban/rural environment. Supplementary Table S10. The food preparation habits assessed while using Adolescents’ Food Habits Checklist (AFHC) in the period during the COVID-19 pandemic, as declared by the adolescents from the Diet and Activity of Youth during COVID-19 (DAY-19) Study cohort (*n* = 1333), stratified by urban/rural environment. Supplementary Table S11. The food consumption habits assessed while using Adolescents’ Food Habits Checklist (AFHC) in the period before the COVID-19 pandemic, as declared by the adolescents from the Diet and Activity of Youth during COVID-19 (DAY-19) Study cohort (*n* = 1333), stratified by urban/rural environment. Supplementary Table S12. The food consumption habits assessed while using Adolescents’ Food Habits Checklist (AFHC) in the period during the COVID-19 pandemic, as declared by the adolescents from the Diet and Activity of Youth during COVID-19 (DAY-19) Study cohort (*n* = 1333), stratified by urban/rural environment.
