# Supplementary material for: Analysis of Food Habits during Pandemic in a Polish Population-Based Sample of Primary School Adolescents: Diet and Activity of Youth during COVID-19 (DAY-19) Study
Source: Nutrients. 2021 Oct 22;13(11):3711. doi: 10.3390/nu13113711 (PMC8622037; doi:10.3390/nu13113711)
Supplement: Supplementary file 1 [file nutrients-13-03711-s001.zip › nutrients-1392112-supplementary.pdf]

nutrients

Supplementary Materials

Analysis of Food Habits during Pandemic in a Polish Population-Based Sample of Primary School Adolescents: Diet and Activity of Youth During COVID-19 (DAY-19) Study

Aleksandra Kołota * and Dominika Głąbska

Department of Dietetics, Institute of Human Nutrition Sciences, Warsaw University of Life Sciences (SGGW-WULS), 159c Nowoursynowska Street, 02-776 Warsaw, Poland; dominika_glabska@sggw.edu.pl (D.G.)
* Correspondence: aleksandra_kolota@sggw.edu.pl; Tel.: +48-22-5937186

Table S1. The food purchase habits assessed while using Adolescents' Food Habits Checklist (AFHC) in the period before the COVID-19 pandemic, as declared by the adolescents from the Diet and Activity of Youth during COVID-19 (DAY-19) Study cohort (n = 1,333), stratified by gender.

Food purchase habits assessed within AFHC*		Girls (n=710)	Boys (n=623)	*p***
If I am having lunch away from home, I often choose a low-fat option	True	302 (42.5%)	236 (37.9%)	0.1023
	False	210 (29.6%)	182 (29.2%)	
	Not applicable	198 (27.9%)	205 (32.9%)	
If I am buying crisps, I often choose a low-fat brand	True	151 (21.3%)	150 (24.1%)	0.2405
	False	425 (59.9%)	374 (60.0%)	
	Not applicable	134 (18.8%)	99 (15.9%)	
I often buy pastries or cakes	True	261 (36.8%)	188 (30.2%)	0.0112
	False	449 (36.2%)	435 (69.8%)	
I rarely eat takeaway meals	True	581 (81.8%)	504 (80.9%)	0.6629
	False	129 (18.2%)	119 (19.1%)	
When I am buying a soft drink, I usually choose a diet drink	True	285 (40.1%)	196 (31.5%)	0.0009
	False	425 (59.9%)	427 (68.5%)	
If I am having a dessert or pudding in a restaurant, I usually choose the healthiest one	True	117 (16.5%)	97 (15.6%)	0.2319
	False	319 (44.9%)	257 (41.2%)	
	Not applicable	274 (38.6%)	269 (43.2%)	

* AFHC – Adolescents' Food Habits Checklist [37]; ** chi^2 test.

Table S2. The food purchase habits assessed while using Adolescents' Food Habits Checklist (AFHC) in the period during the COVID-19 pandemic, as declared by the adolescents from the Diet and Activity of Youth during COVID-19 (DAY-19) Study cohort (n = 1,333), stratified by gender.

Food purchase habits assessed within AFHC*		Girls (n=710)	Boys (n=623)	*p***
If I am having lunch away from home, I often choose a low-fat option	True	253 (35.6%)	203 (32.6%)	0.3161
	False	196 (27.6%)	169 (27.1%)	
	Not applicable	261 (36.8%)	254 (59.7%)	
If I am buying crisps, I often choose a low-fat brand	True	170 (23.9%)	161 (25.8%)	0.0175
	False	372 (52.4%)	354 (56.8%)	
	Not applicable	168 (23.7%)	108 (17.4%)	
I often buy pastries or cakes	True	163 (22.9%)	159 (25.5%)	0.2751
	False	547 (77.1%)	464 (74.5%)	
I rarely eat takeaway meals	True	597 (84.1%)	511 (82.0%)	0.3159
	False	113 (15.9%)	112 (18.0%)	
When I am buying a soft drink, I usually choose a diet drink	True	318 (44.8%)	212 (34.0%)	0.0001
	False	392 (55.2%)	411 (66.0%)	
If I am having a dessert or pudding in a restaurant, I usually choose the healthiest one	True	127 (17.9%)	99 (15.9%)	0.3360
	False	291 (40.9%)	244 (39.2%)	
	Not applicable	292 (41.2%)	280 (44.9%)	

* AFHC – Adolescents' Food Habits Checklist [37]; ** chi^2 test.

Table S3. The food preparation habits assessed while using Adolescents' Food Habits Checklist (AFHC) in the period before the COVID-19 pandemic, as declared by the adolescents from the Diet and Activity of Youth during COVID-19 (DAY-19) Study cohort (n = 1,333), stratified by gender.

Food preparation habits assessed within AFHC*		Girls (n=710)	Boys (n=623)	*p***
I usually avoid eating fried foods	True	305 (42.9%)	204 (32.7%)	0.0001
	False	405 (57.1%)	419 (67.3%)	
I try to keep my overall fat intake down	True	404 (56.9%)	320 (51.4%)	0.0429
	False	306 (43.1%)	303 (48.6%)	
I try to keep my overall sugar intake down	True	399 (56.2%)	342 (54.9%)	0.6330
	False	311 (43.8%)	281 (45.1%)	
If I am having a dessert at home, I try to have something low in fat	True	231 (32.5%)	202 (32.4%)	0.7929
	False	357 (50.3%)	322 (51.7%)	
	Not applicable	122 (17.2%)	99 (15.9%)	
I usually eat at least one serving of vegetables (excluding potatoes) or salad with my evening meal	True	528 (74.4%)	478 (76.7%)	0.3178
	False	182 (25.6%)	145 (23.3%)	
When I put butter or margarine on bread, I usually spread it thinly	True	485 (68.3%)	431 (69.2%)	0.0134
	False	92 (12.9%)	106 (17.0%)	
	Not applicable	133 (18.8%)	86 (13.8%)	
If I have a packed lunch, I usually include some chocolate and/or biscuits	True	253 (35.6%)	182 (29.2%)	0.0305
	False	387 (54.5%)	364 (58.4%)	
	Not applicable	70 (9.9%)	77 (12.4%)	
I often have cream on desserts	True	154 (21.7%)	134 (21.5%)	0.0937
	False	484 (68.2%)	402 (64.5%)	
	Not applicable	72 (10.1%)	87 (14.0%)	

* AFHC – Adolescents' Food Habits Checklist [37]; ** chi^2 test.

Table S4. The food preparation habits assessed while using Adolescents' Food Habits Checklist (AFHC) in the period during the COVID-19 pandemic, as declared by the adolescents from the Diet and Activity of Youth during COVID-19 (DAY-19) Study cohort (n = 1,333), stratified by gender.

Food preparation habits assessed within AFHC*		Girls (n=710)	Boys (n=623)	p**
I usually avoid eating fried foods	True	334 (47.0%)	202 (32.4%)	1.0000
	False	376 (53.0%)	421 (67.6%)	
I try to keep my overall fat intake down	True	463 (65.2%)	337 (54.1%)	<0.0001
	False	247 (34.8%)	286 (45.9%)	
I try to keep my overall sugar intake down	True	461 (64.9%)	461 (73.9%)	0.6444
	False	249 (35.1%)	262 (26.1%)	
If I am having a dessert at home, I try to have something low in fat	True	269 (37.9%)	214 (34.3%)	0.0846
	False	322 (45.3%)	320 (51.4%)	
	Not applicable	119 (16.8%)	89 (14.3%)	
I usually eat at least one serving of vegetables (excluding potatoes) or salad with my evening meal	True	576 (81.1%)	488 (78.3%)	0.2043
	False	134 (18.9%)	135 (21.7%)	
When I put butter or margarine on bread, I usually spread it thinly	True	478 (67.3%)	434 (69.7%)	0.0008
	False	86 (12.1%)	104 (16.7%)	
	Not applicable	146 (20.6%)	85 (13.6%)	
If I have a packed lunch, I usually include some chocolate and/or biscuits	True	163 (22.9%)	145 (23.3%)	0.9734
	False	423 (59.6%)	372 (59.7%)	
	Not applicable	124 (17.5%)	106 (17.0%)	
I often have cream on desserts	True	149 (21.0%)	142 (22.8%)	0.0789
	False	481 (67.7%)	389 (62.4%)	
	Not applicable	80 (11.3%)	92 (14.8%)	

* AFHC – Adolescents' Food Habits Checklist [37]; ** chi^2 test.

Table S5. The food consumption habits assessed while using Adolescents' Food Habits Checklist (AFHC) in the period before the COVID-19 pandemic, as declared by the adolescents from the Diet and Activity of Youth during COVID-19 (DAY-19) Study cohort (n = 1,333), stratified by gender.

Food consumption habits assessed within AFHC*		Girls (n=710)	Boys (n=623)	p**
I usually eat a dessert or pudding if there is one available	True	505 (71.1%)	448 (71.9%)	0.7518
	False	205 (28.9%)	175 (28.1%)	
I make sure I eat at least one serving of fruit a day	True	587 (82.7%)	510 (81.9%)	0.6976
	False	123 (17.3%)	113 (18.1%)	
I avoid eating lots of sausages and burgers	True	417 (58.7%)	366 (58.7%)	1.0000
	False	150 (21.1%)	193 (31.0%)	
	Not applicable	143 (20.2%)	64 (10.3%)	
I make sure I eat at least one serving of vegetables or salad a day	True	500 (70.4%)	449 (72.1%)	0.5076
	False	210 (29.6%)	174 (27.9%)	
I try to ensure I eat plenty of fruit and vegetables	True	564 (79.4%)	445 (71.4%)	0.0007
	False	146 (20.6%)	178 (28.6%)	
I often eat sweet snacks between meals	True	338 (47.6%)	285 (45.7%)	0.4972
	False	372 (52.4%)	338 (54.3%)	
When I have a snack between meals, I often choose fruit	True	335 (47.2%)	276 (44.3%)	0.0506
	False	287 (40.4%)	271 (43.5%)	
	Not applicable	88 (12.4%)	76 (12.2%)	
I eat at least three servings of fruit most days	True	341 (48.0%)	291 (46.7%)	0.6308
	False	369 (52.0%)	332 (53.3%)	
I generally try to have a healthy diet	True	513 (72.2%)	450 (72.2%)	1.0000
	False	197 (27.8%)	173 (27.8%)	

* AFHC – Adolescents' Food Habits Checklist [37]; ** chi^2 test.

Table S6. The food consumption habits assessed while using Adolescents' Food Habits Checklist (AFHC) in the period during the COVID-19 pandemic, as declared by the adolescents from the Diet and Activity of Youth during COVID-19 (DAY-19) Study cohort (n = 1,333), stratified by gender.

Food consumption habits assessed within AFHC*		Girls (n=710)	Boys (n=623)	*p***
I usually eat a dessert or pudding if there is one available	True	505 (71.5%)	458 (73.5%)	0.3312
	False	205 (28.5%)	165 (26.5%)	
I make sure I eat at least one serving of fruit a day	True	632 (89.0%)	519 (83.3%)	0.0025
	False	78 (11.0%)	104 (16.7%)	
I avoid eating lots of sausages and burgers	True	435 (61.3%)	374 (60.0%)	1.0000
	False	131 (18.4%)	183 (29.4%)	
	Not applicable	144 (20.3%)	66 (10.6%)	
I make sure I eat at least one serving of vegetables or salad a day	True	573 (80.7%)	472 (75.8%)	0.0297
	False	137 (19.3%)	151 (24.2%)	
I try to ensure I eat plenty of fruit and vegetables	True	622 (87.6%)	474 (76.1%)	1.0000
	False	88 (12.4%)	149 (23.9%)	
I often eat sweet snacks between meals	True	322 (45.3%)	319 (51.2%)	0.0329
	False	388 (54.7%)	304 (48.8%)	
When I have a snack between meals, I often choose fruit	True	389 (54.8%)	297 (47.7%)	0.0131
	False	235 (33.1%)	254 (40.8%)	
	Not applicable	86 (12.1%)	72 (11.5%)	
I eat at least three servings of fruit most days	True	420 (59.1%)	334 (53.6%)	0.0416
	False	290 (40.9%)	289 (46.4%)	
I generally try to have a healthy diet	True	593 (83.5%)	475 (76.2%)	0.0009
	False	117 (16.5%)	148 (23.8%)	

* AFHC – Adolescents' Food Habits Checklist [37]; ** chi^2 test.

Table S7. The food purchase habits assessed while using Adolescents' Food Habits Checklist (AFHC) in the period before the COVID-19 pandemic, as declared by the adolescents from the Diet and Activity of Youth during COVID-19 (DAY-19) Study cohort (n = 1,333), stratified by urban/rural environment.

Food purchase habits assessed within AFHC*		Urban (n=973)	Rural (n=360)	*p***
If I am having lunch away from home, I often choose a low-fat option	True	392 (40.3%)	146 (40.5%)	0.3977
	False	278 (28.6%)	114 (31.6%)	
	Not applicable	303 (31.1%)	100 (27.9%)	
If I am buying crisps, I often choose a low-fat brand	True	216 (22.2%)	85 (23.6%)	0.4398
	False	593 (60.9%)	206 (57.2%)	
	Not applicable	164 (16.9%)	69 (19.2%)	
I often buy pastries or cakes	True	322 (33.1%)	127 (35.3%)	0.4538
	False	651 (66.9%)	233 (64.7%)	
I rarely eat takeaway meals	True	785 (80.7%)	300 (83.3%)	0.2688
	False	188 (19.3%)	60 (16.7%)	
When I am buying a soft drink, I usually choose a diet drink	True	333 (34.2%)	148 (41.1%)	0.0166
	False	640 (65.8%)	212 (58.9%)	
If I am having a dessert or pudding in a restaurant, I usually choose the healthiest one	True	154 (15.8%)	60 (16.7%)	0.0429
	False	440 (45.2%)	136 (37.8%)	
	Not applicable	379 (30.0%)	164 (45.5%)	

* AFHC – Adolescents' Food Habits Checklist [37]; ** chi^2 test.

Table S8. The food purchase habits assessed while using Adolescents' Food Habits Checklist (AFHC) in the period during the COVID-19 pandemic, as declared by the adolescents from the Diet and Activity of Youth during COVID-19 (DAY-19) Study cohort (n = 1,333), stratified by urban/rural environment.

Food purchase habits assessed within AFHC*		Urban (n=973)	Rural (n=360)	*p***
If I am having lunch away from home, I often choose a low-fat option	True	322 (33.1%)	131 (36.4%)	0.2967
	False	277 (28.5%)	88 (24.4%)	
	Not applicable	374 (38.4%)	141 (39.2%)	
If I am buying crisps, I often choose a low-fat brand	True	236 (24.2%)	95 (26.4%)	0.1561
	False	545 (56.0%)	181 (50.3%)	
	Not applicable	192 (19.8%)	84 (76.7%)	
I often buy pastries or cakes	True	233 (24.0%)	89 (24.7%)	0.7693
	False	740 (76.0%)	271 (75.3%)	
I rarely eat takeaway meals	True	803 (82.5%)	305 (84.7%)	0.3425
	False	170 (17.5%)	55 (15.3%)	
When I am buying a soft drink, I usually choose a diet drink	True	368 (37.8%)	162 (45.0%)	0.0174
	False	605 (62.2%)	198 (55.0%)	
If I am having a dessert or pudding in a restaurant, I usually choose the healthiest one	True	162 (16.6%)	64 (17.8%)	0.0222
	False	412 (42.3%)	123 (34.2%)	
	Not applicable	399 (41.1%)	173 (48.0%)	

* AFHC – Adolescents' Food Habits Checklist [37]; ** chi^2 test.

Table S9. The food preparation habits assessed while using Adolescents' Food Habits Checklist (AFHC) in the period before the COVID-19 pandemic, as declared by the adolescents from the Diet and Activity of Youth during COVID-19 (DAY-19) Study cohort (n = 1,333), stratified by urban/rural environment.

Food preparation habits assessed within AFHC*		Urban (n=973)	Rural (n=360)	*p***
I usually avoid eating fried foods	True	365 (37.5%)	144 (40.0%)	0.4065
	False	608 (62.5%)	216 (60.0%)	
I try to keep my overall fat intake down	True	528 (54.3%)	196 (54.4%)	0.9563
	False	445 (45.7%)	164 (45.6%)	
I try to keep my overall sugar intake down	True	532 (54.7%)	209 (58.0%)	0.2701
	False	441 (45.3%)	151 (42.0%)	
If I am having a dessert at home, I try to have something low in fat	True	303 (31.1%)	130 (36.1%)	0.1668
	False	510 (52.4%)	169 (46.9%)	
	Not applicable	160 (16.5%)	61 (17.0%)	
I usually eat at least one serving of vegetables (excluding potatoes) or salad with my evening meal	True	728 (74.8%)	278 (77.2%)	0.3655
	False	245 (25.2%)	82 (22.8%)	
When I put butter or margarine on bread, I usually spread it thinly	True	653 (67.1%)	263 (73.0%)	0.0618
	False	157 (16.1%)	41 (11.4%)	
	Not applicable	163 (16.8%)	56 (15.6%)	
If I have a packed lunch, I usually include some chocolate and/or biscuits	True	324 (33.3%)	111 (30.9%)	0.0638
	False	532 (54.7%)	219 (60.8%)	
	Not applicable	117 (12.0%)	30 (8.3%)	
I often have cream on desserts	True	210 (21.6%)	78 (21.7%)	0.7294
	False	651 (66.9%)	235 (65.3%)	
	Not applicable	112 (11.5%)	47 (13.0%)	

* AFHC – Adolescents' Food Habits Checklist [37]; ** chi^2 test.

Table S10. The food preparation habits assessed while using Adolescents' Food Habits Checklist (AFHC) in the period during the COVID-19 pandemic, as declared by the adolescents from the Diet and Activity of Youth during COVID-19 (DAY-19) Study cohort (n = 1,333), stratified by urban/rural environment.

Food preparation habits assessed within AFHC*		Urban (n=973)	Rural (n=360)	*p***
I usually avoid eating fried foods	True	387 (39.8%)	149 (41.4%)	0.5934
	False	586 (60.2%)	211 (58.6%)	
I try to keep my overall fat intake down	True	577 (59.3%)	223 (61.9%)	0.3818
	False	396 (40.7%)	137 (38.1%)	
I try to keep my overall sugar intake down	True	588 (60.4%)	234 (65.0%)	0.1277
	False	385 (39.6%)	126 (35.0%)	
If I am having a dessert at home, I try to have something low in fat	True	344 (35.4%)	139 (38.6%)	0.4269
	False	479 (49.2%)	163 (45.3%)	
	Not applicable	150 (15.4%)	58 (16.1%)	
I usually eat at least one serving of vegetables (excluding potatoes) or salad with my evening meal	True	773 (79.4%)	291 (80.8%)	0.5752
	False	200 (20.6%)	69 (19.2%)	
When I put butter or margarine on bread, I usually spread it thinly	True	659 (67.7%)	253 (70.3%)	0.2573
	False	148(15.2%)	42 (11.7%)	
	Not applicable	166 (17.1%)	65 (18.0%)	
If I have a packed lunch, I usually include some chocolate and/or biscuits	True	234 (24.0%)	74 (20.6%)	0.0233
	False	559 (57.5%)	236 (65.5%)	
	Not applicable	180 (18.5%)	50 (13.9%)	
I often have cream on desserts	True	214 (21.9%)	77 (21.4%)	0.8033
	False	637 (65.5%)	233 (64.7%)	
	Not applicable	122 (12.6%)	50 (13.9%)	

* AFHC – Adolescents' Food Habits Checklist [37]; ** chi^2 test.

Table S11. The food consumption habits assessed while using Adolescents' Food Habits Checklist (AFHC) in the period before the COVID-19 pandemic, as declared by the adolescents from the Diet and Activity of Youth during COVID-19 (DAY-19) Study cohort (n = 1,333), stratified by urban/rural environment.

Food consumption habits assessed within AFHC*		Urban (n=973)	Rural (n=360)	*p***
I usually eat a dessert or pudding if there is one available	True	689 (70.8%)	264 (73.3%)	0.3652
	False	284 (29.2%)	96 (26.7%)	
I make sure I eat at least one serving of fruit a day	True	797 (81.9%)	300 (83.3%)	0.5457
	False	176 (18.1%)	60 (16.7%)	
I avoid eating lots of sausages and burgers	True	557 (57.2%)	226 (62.8%)	0.0844
	False	253 (26.0%)	90 (25.0%)	
	Not applicable	163 (16.8%)	44 (12.2%)	
I make sure I eat at least one serving of vegetables or salad a day	True	691 (71.0%)	258 (71.7%)	0.8162
	False	282 (29.0%)	102 (28.2%)	
I try to ensure I eat plenty of fruit and vegetables	True	728 (74.8%)	281 (78.0%)	0.2214
	False	245 (25.2%)	79 (22.0%)	
I often eat sweet snacks between meals	True	461 (47.4%)	162 (45.0%)	0.4393
	False	512 (52.6%)	198 (55.0%)	
When I have a snack between meals, I often choose fruit	True	434 (44.6%)	177 (49.2%)	0.3310
	False	417 (42.8%)	141 (39.2%)	
	Not applicable	122 (12.6%)	42 (11.6%)	
I eat at least three servings of fruit most days	True	436 (44.8%)	196 (54.4%)	0.0018
	False	537 (55.2%)	164 (45.6%)	
I generally try to have a healthy diet	True	706 (72.5%)	257 (71.4%)	0.6722
	False	267 (27.5%)	103 (28.6%)	

* AFHC – Adolescents' Food Habits Checklist [37]; ** chi^2 test.

Table S12. The food consumption habits assessed while using Adolescents' Food Habits Checklist (AFHC) in the period during the COVID-19 pandemic, as declared by the adolescents from the Diet and Activity of Youth during COVID-19 (DAY-19) Study cohort (n = 1,333), stratified by urban/rural environment.

Food consumption habits assessed within AFHC*		Urban (n=973)	Rural (n=360)	*p***
I usually eat a dessert or pudding if there is one available	True	702 (72.4%)	261 (72.5%)	0.8993
	False	271 (27.6%)	99 (27.5%)	
I make sure I eat at least one serving of fruit a day	True	830 (85.3%)	321 (89.2%)	0.0681
	False	143 (14.7%)	39 (10.8%)	
I avoid eating lots of sausages and burgers	True	577 (59.3%)	232 (64.4%)	0.1871
	False	234 (24.0%)	80 (22.2%)	
	Not applicable	162 (16.7%)	48 (13.4%)	
I make sure I eat at least one serving of vegetables or salad a day	True	747 (76.8%)	298 (82.8%)	0.0180
	False	226 (23.2%)	62 (17.2%)	
I try to ensure I eat plenty of fruit and vegetables	True	788 (81.0%)	308 (85.5%)	0.0527
	False	185 (19.0%)	52 (14.5%)	
I often eat sweet snacks between meals	True	466 (47.9%)	175 (48.6%)	0.8162
	False	507 (52.1%)	185 (51.4%)	
When I have a snack between meals, I often choose fruit	True	482 (49.5%)	204 (56.7%)	0.0678
	False	372 (38.2%)	117 (32.5%)	
	Not applicable	119 (12.3%)	39 (10.8%)	
I eat at least three servings of fruit most days	True	525 (53.9%)	229 (63.6%)	0.0016
	False	448 (46.1%)	131 (36.4%)	
I generally try to have a healthy diet	True	770 (79.1%)	298 (82.8%)	0.1392
	False	203 (20.9%)	62 (17.2%)	

* AFHC – Adolescents' Food Habits Checklist [37]; ** chi^2 test.
